# Effect of Land Expropriation on Land-Lost Farmers’ Health: Empirical Evidence from Rural China

**DOI:** 10.3390/ijerph16162934

**Published:** 2019-08-15

**Authors:** Yuxin Wang, Wenlong Li, Jinping Xiong, Ying Li, Huaqing Wu

**Affiliations:** 1 School of Economics, Hefei University of Technology, Hefei 230601, China; 2 Center for Industrial information and Economic Research, Hefei University of Technology, Hefei 230601, China

**Keywords:** land expropriation, land-lost farmers, health

## Abstract

With rapid urbanization and industry development, China has witnessed substantial land acquisition. Using the rural household survey data, this paper examines the impact of land expropriation on land-lost farmers’ self-reported health with the ordered probit model and investigates the possible mechanisms. The results show that the land expropriation puts higher health risks over those land-lost farmers and the health status of land-lost farmers is significantly worse than that of those with land. Land expropriation has a negative impact on the land-lost farmer’s health through income effects and psychological effects. The health status of land-lost farmers can be enhanced through amending current land requisition policies, increasing the amount of compensation, improving the earning capacity of land-lost farmers and strengthening mental health education.

## 1. Introduction

With the advancement and acceleration of industrialization and urbanization in China, the expropriation of land caused a large number of farmers to leave their land and become the land-lost farmers. Statistics show that there are 50 million land-lost farmers after land expropriation in China, and about 30 million land-lost farmers’ living standards have been greatly declined [1,2]. After the expropriation of farmers’ land, the land-lost farmers face pressure from the economic model, social interaction and other aspects of change and re-adaptation requirements, which may have an impact on the health of the farmers, including physical and mental health. Health, in turn, affects individual behavior and health can affect both individual productivity and output [3]. Farmers with poor health are more likely to end up in poverty [4,5].

The existing literature mainly focused on the issues of pension system and income for land-lost farmers. However, the research on whether land expropriation has any effect on the health of farmers is still relatively rare. Therefore, we would like to know if there is any health risk of land-lost farmers under the background of urbanization. If so, what are the health effects of land expropriation on rural residents? Are these effects positive or negative? What are the formation mechanisms and the channels of influence? Is the influence on the farmers with different gender and socio-economic status consistent? How can the health safeguard system be effectively built for land-lost farmers? Obviously, answering these questions is the main purpose of this research. We can find the key points for improving the health of land-lost farmers through targeted intervention of channels, which is of great theoretical and practical significance for the formulation of a reasonable agricultural land compensation system, the implementation of the Rural Vitalization Strategy and the beautiful countryside construction in China.

Among the existing literature, few studies have quantitatively examined the link between land-lost and the health status of the farmers at the local level, largely due to the lack of detailed information and micro data. This work adds to the literature in the following ways. Firstly, the previous studies focus on qualitative description and case analysis, while the empirical analysis based on micro-data is still scarce. We conducted a rigorous empirical study based on the survey data of rural households in Chengdu in 2011 and add to the growing empirical literature examining the relationship between land expropriation and land-lost farmers’ health at the micro-level. Secondly, although the influence of land expropriation on the farmer’s health is considered in previous studies, this study provides the evidence to explore the issue from a new perspective: that of considering investigating this complex mechanism by income and psychological effects. Our results provide new insights on the issue of Chinese rural land expropriation. Our study consequently calls for reinforced actions from the Chinese public authorities in order to improve the well-being of the land-lost farmers. Thirdly, existing empirical studies on the effect of land expropriation on land-lost farmer’s health adopted the simple OLS (Ordinary Least Square) and probit model. We adopt an ordered probit model, so as to more accurately identify the health effects of land-lost farmers.

The remainder of the paper is organized as follows. In Section 2 we review the related literature. In Section 3 we introduce the data source, the definition of related variables and the construction of the model. The empirical results are presented in Section 4. We make a further discussion on the formation mechanism of the health effects of land-lost farmers in Section 5. The last section summarizes the main conclusions.

## 2. Literature Review

There is a large literature base regarding the influence factors which affect residents’ health. Most of the literature on residents’ health are based on the theory of the demand for health (Grossman, 1972) to explore the determinants of health from dimensions like age, gender, income level and social capital, etc. [6,7,8,9,10,11,12,13,14]. At the micro level, age, gender, educational level, marital status, environmental quality, water quality, sanitation level, income level and religious beliefs all have an impact on the health of rural residents [11,12,13,14,15,16,17]. At the macro level, health expenditure in rural areas, accessibility of medical services and outflow of rural labor force are closely related to the health of the rural residents [18,19,20].

However, for a long time, there was less attention paid to whether the land expropriation will affect the health of farmers. The reason is mostly due to the lack of detailed survey data. Jacobs (2004) and Marco-Thyse (2006) found that land expropriation by urbanization in African countries reduced the health status of farmers [21,22]. In the opinion of Summerfield (2007) and Friedman (2009), improving the health status of land-lost farmers requires improvements in the medical care and retirement benefits of land-lost farmers [23,24]. Fearnside (2001) and Campbell et al. (2010) claimed that land expropriation could lead to a decline in the health of land-lost farmers and cause social instability [25,26]. Yang (2017) found that people who lived in the communities with land expropriation had 0.75 units lower depression score [27]. Wu et al. (2009) studied the health status of land-lost farmers in northern Jiangsu and found that the health problems were serious [28]. Qin et al. (2012) found that urbanization has reduced the health of land-lost farmers based on the probit model [1]. Yu (2012) argued that psychological gaps and imbalances are more likely to breed among land-lost farmers and induce their health risks [29].

To sum up, the association between land expropriation and health has been less discussed, and we do not clearly know whether the effect of land expropriation on health exists in rural China. Under the background of rapid economic development and urbanization, rural land expropriation will become more and more common in China, thus, the association between land expropriation and health is worthy of discussion. This study aims to address this research gap. It is hoped that these results provide new evidence for the issue on the land-lost farmers’ health, and enables us to better provide new ideas for the improvement of relevant policies after land expropriation.

## 3. Data, Variables and Model Specification

### 3.1. Data

The data set used for the analysis comes from the 2011 rural household survey data in Chengdu. The survey conducted in 2011 from June to September and there was no further investigation. The data were created in the frame of World Bank and Chinese Academy of Social Sciences funded projects. With the advancement of urbanization, the scale of land expropriation in Chengdu is gradually increasing. By June 2015, a total of 0.0746 million hectare of farmland was expropriated and about 637,000 farmers lost their land in whole or in part [30].

Multi-stage sampling method was used in the survey. In the first phase of the survey, methods sampling proportioned to the scale was taken. Three surveyed counties were randomly selected from Chengdu. In each county, three townships were randomly selected and three administrative villages were selected from each township. Finally, household surveys were conducted on rural households according to a combination of random sampling and typical sampling in administrative villages. Missing or incomplete information questionnaires were eliminated, and the samples of students in school and the samples of farmers who lost their working capability entirely by illness or disability were deleted. In this study, missing data in land expropriation, health, hukou and age variables were omitted by a listwise deletion strategy. After data processing, a total of 3945 rural residents were used as research samples, of which 1199 were rural land-lost farmers, accounting for 30.39% of the total sample.

### 3.2. Variable Definition and Measurement

#### 3.2.1. Dependent Variable

Quantifying health status is always a challenging problem. Self-reported health status as an indicator of personal health may be subjectively influenced by individual respondents. But the main advantage of self-reported health is that it can fully reflect the physiological and mental health of individuals. It was widely used in the previous literature and many studies confirmed the rationality of this indicator [8,13,14,31,32,33,34,35,36,37]. This paper also used self-reported health status as the primary health indicator.

Self-reported health was collected during the main interview. The respondents were asked to rate their own general health on a five-point Likert scale: Would you say your health is very good, good, fair, poor or very poor? The answers were coded as (1) “Very Poor”, (2) “Poor”, (3) “Fair”, (4) “Good”, (5) “Very Good”.

#### 3.2.2. The Explanatory Variable and Control Variables

Whether the farmers loss their land or not was the main explanatory variable. The main explanatory variable was a dummy variable indicating the status of the land expropriation; that is, the dichotomous variables took the value one if the farmers have lost all or part of the land and took the value zero if they have not lost their land. Most of the farmers lost their land 2 to 4 years ago. The mode of compensation was the way for land expropriation and the government does not provide any jobs for the land-lost farmers. Therefore, this survey provided us a good source of data to discover the effect of land expropriation on the health of rural residents.

We controlled for other factors that may affect the individual’s health, mainly including gender, age, education level and other family factors such as family size, family income per capita and family housing marketing value [6,7,8,12,13,15,16,31].

Table 1 provides an overview and descriptive statistics of the variables used in the empirical analysis. The Spearman correlation matrix of all variables are shown in Table S1 in the supplementary material.

From Table 1, we can see that the average value of self-reported health was 3.823, which reflects that the self-reported health of rural residents was between “fair” and “good”, which was consistent with the previous literature on the health of rural residents [37]. The average value of land lost was 0.304, which shows that land-lost farmers accounted for about 1/3 of the total sample. The proportion of females was 49.39%, which is consistent with the demographic characteristics of females in China that are less than males. The average age of rural residents surveyed was 45.01 years old and the average education years was 7.992 years. The average family size of respondents was 4.116.

### 3.3. Model Specification

According to Grossman’s theory, influencing factors such as the age, education level and gender of residents affect the health status of residents. Since health was a categorical variable in this study, and was ranked and discontinuous, the ordinary least squares (OLS) is not suitable for estimate the equation. Therefore, we adopted the ordered probit model to examine the impact of land expropriation on land-lost farmers’ self-reported health.

Following the literature [37,38,39], our general estimation approach is as seen as follows:

(1) $$Health_{i}=F(\lambda Landlost_{i}+\beta X_{i}+\mu_{i})$$

The dependent variable, $Health_{i}$ is the self-reported health status of rural residents. Self-reported health, a widely used and validated measure of general health, was assessed by the following question: “Would you say your health is very good, good, fair, poor or very poor?” The question has five answer options including (1) “Very Poor”, (2) “Poor”, (3) “Fair”, (4) “Good”, (5) “Very Good”. It is widely used in the previous literature and many studies confirm the rationality of this indicator [31,32,33,34,35,36,37]. $Landlost_{i}$ is the core explanatory variable. It is a dummy variable, taking the value 1 when the farmer lost his land, and 0 otherwise. $X_{i}$ represents the set of control variables that reflect the characteristics of individuals and families, mainly including the gender, age, marital status, family size and per capita income of rural residents. F(⋅) is a non-linear function, and the specific form is:

(2) $$F(Y_{i}^{*})=\left\{ \begin{array}{cc} 1 & Y_{i}^{*}\leq r_{1}\\ 2 & r_{1}<Y_{i}^{*}\leq r_{2}\\ \vdots & \vdots \\ J & r_{j-1}\leq Y_{i}^{*} \end{array}\right.$$

In this model, $Y_{i}^{*}$ is the unobservable latent variable, $r_{1}\;<\;r_{2}\;<\;r_{3}\;<\;\cdots <\;r_{J-1}$, is the estimated parameter and called the tangent point.

## 4. Econometric Analysis Results

Firstly, we studied whether the farmer losing their land could affect their health status. The analysis was performed based on an ordered probit model. Secondly, margin effects were analyzed. Lastly, we tested the robustness of our results.

### 4.1. Model Estimation Results

Table 2 presents estimates of the health Equation (1). We mainly focused on the effect of land expropriation on the health status of rural households. Model 1 only controlled the basic characteristics of individuals such as gender, age, educational level and marital status. Model 2 reported the coefficients for adding other control variables such as medical expenses last year and participation status in the New Rural Cooperative Medical Scheme. Model 3 reported the estimated coefficients for a full set of control variables.

Models (1) to (3) have the same estimation results. The results revealed a negative and significant effect of land expropriation on self-reported health. This means that the land expropriation put higher health risks over those land-lost farmers and the health status of land-lost farmers was significantly worse than that of those being with land. Land expropriation had a negative impact on the land-lost farmer’s health. In column four of Table 2, we re-estimated with the ordered logit model. The results for the main effect were very similar to the results with the ordered probit model.

Regarding the control variables, age was significantly negative at the 1% level, hukou and medical expenses were significantly negative at the 1% level. Gender was significantly positive at the 1% level; education level and family income per capita were significantly positive at the 1% level; family size and family house marketing value were significantly positive at the 5% level. The regression results of gender showed that males had far better health than females. Age was negatively correlated with health. The educational level, the family size and the family per capita income will improve the health status of the farmers.

### 4.2. Marginal Effects Analysis

The marginal effects of the explanatory variable and other control variables form ordered probit model is presented in Table 3.

The marginal effects showed that under the condition of controlling other variables farmers who lost their land were more likely to report poor self-rated health. Specifically, land expropriation reduced the probability that the farmer self-rated health “very good” by 3.847%, and the probability of “good” decreased by 0.211%. The probability of “Fair” increased by 2.117%, the probability of “poor” increased by 1.726%, and the probability of “very bad” increased by 0.225%.

The marginal effects of other control variables are consistent with the expected. For example, for each ten thousand yuan increase in per capita household income, the probabilities of “very poor”, “poor” and “fair” self-rated health dropped by 0.249%, 1.978% and 2.516% respectively; and the probability of “good”, “very good” self-rated health rose by 0.167% and 4.576%. Education was positively correlated with self-reported health and age was negatively correlated with health. With increasing age one year, the probabilities of “very poor”, “poor” and “fair” self-rated health rose by 0.04%, 0.31% and 0.39% respectively; and the probability of “good”, “very good” self-rated health dropped 0.03% and 0.71%.

### 4.3. Robustness Checks

In this section, we investigated the robustness of our results in the following several aspects.

(1) We considered adding some other control variables. We conducted sensitivity checks by adding other control variables that may also affect the health status of the farmers, including an indicator of whether a farmer was engaged in business the past year (Businesse), the expenditures on nutrition and health care (Expendnutri) and a dummy variable, whether a farmer is a China Communist Party member (Communistpm). Adding the control variables had little effect on the effect of the land expropriation. The coefficients of the control variables were all significant and had expected signs. More importantly, our main results were unchanged and the coefficient estimates varied little with our choice about whether to include other control variables or not from Table 4. In the following regressions we only report estimates of the parsimonious specification and omitted the estimated results of the other control variables due to space constraints.

(2) We considered replacing the independent variable. Instead of using an ordered self-rated health, we used a binary variable to reflect the health statues of the farmers. The dichotomous variable took the value one if the farmers considered their health status was good and very good; and took the value zero if they considered their health status was other. We estimated the equation with probit and logit models. The alternative measures confirmed, and even strengthened our previous findings. The results of Model 8 and Model 9 are shown in Table 5.

(3) We considered changing the sample range. We tested whether our results ere sensitive to the sample of the farmers considered in the estimations. First, we excluded the farmers over the age of 65 years, only considering the working-age farmers who are in the 18–65 years range. The third columns (Model 10) in Table 6 present the results of the estimation. The sample restriction had no impact on our previous conclusions. Second, we only considered the sample of rural residents with rural household registration. Model 11 in Table 6 presents these results. Third, farmers were divided into two groups based on the income level of poverty line. According to the poverty line standard set by China in 2011 (the standard of annual per-capita annual net income poverty is 2300 yuan), the total sample was divided into two sub-samples, one being under the poverty line and the other being above the poverty line. As can be seen from the results in the Table 6, land expropriation had a negative effect on the health of farmers with different income levels. Among farmers under the poverty line, the coefficient of land expropriation was −0.123, while the coefficient of the farmers above the poverty line was −0.153. This means that the income effect was more obvious for the poor farmers.

Having experienced the above-mentioned tests, the results were still robust. Land expropriation affected the health status of the rural residents, the health status of land-lost farmers was remarkably lower than that of land-based farmers. Therefore, the results were accepted by the robustness tests, proving their stability and reliability.

## 5. Discussion

The above analysis shows that land expropriation has a significant negative effect on the health status of land-lost farmers. To clarify this relationship, this section we offer the following possible explanations.

### 5.1. Income Effect

The first possible explanation for this effect is that land expropriation can affect farmers’ incomes, resulting in a decline in their health status. Self-reported health of the residents can be improved with increasing income [40,41,42]. When the Chinese government expropriates agricultural land, the compensation policy may be unreasonable. The current land expropriation compensation policy of “multiple output compensation” will lead to the low compensation for land compensation. The compensation is much lower than the market price and the compensation is to be paid to the farmers in a lump sum. This policy led to the farmers losing a long-term source of income and income security. They cannot guarantee the basic living standard of land-lost farmers. In addition, the employment channels of land-lost farmers have changed. The shift from agriculture-related to non-agricultural sectors and the low education level of the land-lost farmers leave the farmers at a lower level in regards to income and obtaining employment. The lower level of income and employment has led to the land-lost farmers not having the necessary resources to devote resources to health-related activities, resulting in the deterioration of the health status of land-lost farmers.

Loss of land may lead to falling the farmers’ income and lack of protection for their long-term livelihoods. Available literature was identified for this review. They found that land expropriation makes the total income of farmers drop by 12.7% and the agricultural-related income by 36.8%; the compensation system of land expropriation damages the basic economy of land-lost farmers rights and interests; the compensation standard is less than 10% of the current land value-added income and the land-lost farmers are not in good condition; more than 90% of land-lost farmers are not satisfied with the compensation standards of local governments [43,44,45,46]. The impact of land expropriation on different incomes of the farmers was used to investigate the income effects in our study.

Table 7 are the results of the land expropriation on the different income of the farmers. Land expropriation will significantly reduce their agriculture-related income and increase the transferred income. But the effect for the non-agricultural income and total income is not significant. The results show that land expropriation had no effects on farmers’ income increase. Therefore, =the compensation should be strengthened for land-lost farmers and the ability to increase the income of the land-lost farmers should be improved, so that farmers have the ability to invest in health.

### 5.2. Psychological Effect

Another possible aspect of land expropriation effects is psychological factors. Firstly, more and more farmers are losing their lands depended upon in the past times. After the land has been expropriated, the land on which generations depended on for survival was lost forever. This event will greatly affect their psychological conditions and cause anxiety in their future lives. This will easily lead to psychological imbalance and increasing insecurity, resulting in health problems. Secondly, the value of the land expropriated has increased substantially after more and more agricultural land has been converted to non-agricultural use. But the land-lost farmers cannot share the benefits brought by land value increment. This will cause a psychological imbalance. In addition, land compensations will be paid in lump sum, resulting in the land-lost farmers’ lack of appropriate social security once the compensation is used up. The land-lost farmers will face the dual pressures of life and employment, likely to cause psychological imbalance and abnormalities. Therefore, land-lost farmers experience higher psychological anomalies which will seriously endanger their health.

Due to the lack of the data, it is impossible to conduct more accurate discrimination on the psychological mechanism of land expropriation, nor can it make any specific contribution to the impact of the land expropriation on the farmer’s mental health. First of all, we provide some indirect evidence to explain the psychological effects. In addition, we examine the effect of land expropriation on land-lost farmers from a gender perspective.

Land-lost farmers’ psychological status showed a trend of deterioration [47]. The overall mental health status of land-lost farmers is not optimistic, exhibiting anxiety, hostility, paranoia and some mental diseases, etc. [48]. More than 80% of the land-lost farmers miss the more primitive way of life previously experienced—“go to work at sunrise and go home at sunset”, and have great concern for their life after land expropriation [29]. All of these studies show that the psychological conditions of farmers have changed after the land expropriation. Different genders may have differences in psychological feelings after losing their land. As can be seen from the Table 8, the effects to males and females are different. The effect of land expropriation to females is significant negative, while to the males is negative, but not significant.

The possible explanation are as follows. On the one hand, the traditional culture of “men go out and women stay home” in China makes women have less social activities than men. Rural women are mostly responsible for cumbersome family affairs and take mental stress more than men. Women are psychologically weak to bear the loss of the land. The worry and anxiety about future life after losing land can easily lead to psychological imbalance and endanger their health. On the other hand, women in rural areas are generally less educated, less able to accept new things, less able to adapt to new situations, less able to cope with stress and mental health. Women tend to grow more anxious than men. They are more severely affected by land expropriation. In addition, women are generally more open and honest when talking about health issues, compared to their male counterparts. They consider more factors than men and will underestimate their own health status.

## 6. Conclusions

This paper, using micro data derived from a questionnaire rural household survey in Chengdu, analyzes whether land expropriation affects the health status of rural residents and investigates the possible mechanisms by ordered probit model, exploring a new research angle. The empirical results demonstrate that land expropriation puts higher health risks over those land-lost farmers and the health status of land-lost farmers is significantly worse than that of those with land. Land expropriation can reduce the probability that the farmer self-rated their health “very good” by 3.847%, the probability of “good” decreases by 0.211% and the probability of “very bad” increases by 0.225%. Land expropriation has a negative impact on the land-lost farmer’s health through income and psychological effects.

These results contribute new evidence to address these questions we proposed before and let us understand the mechanisms of how land expropriation affects the health status of the land-lost farmers. However, given the findings of this study show that land expropriation brings about a worse health status, it is recommended that both compensation-based policies and target-based policies be considered in relation to land expropriation. The rural land in China is bearing the weight of double functions, which are agricultural production and social security. That must be embodied in the compensation. There are some implications for policy that could be extracted from these results. First, the Chinese public authorities may raise the standards for land compensation and resettlement fees for land expropriated according to the social and economic development level. Second, various ways of compensation should be adopted. The mode of compensation depending on the market price and allocation with job should become a new way for land expropriation. Third, it is essential to provide training to land-lost farmers, both to increase their knowledge (so that they can raise their self-health care consciousness), as well as to improve their employment level (so that they can enhance the income-acquiring ability). Accordingly, this study will hopefully encourage further research on health problems in villagers and farmers in developing countries.

Nonetheless, the present study has some limitations. First, this study only used self-reported health to measure health status and ignored other indicators of health, and a comprehensive indicator is more helpful to reveal the secret of land expropriation affecting health. Due to the lack of data, a self-reported health variable can be used to measure health status in the present study. However, numerous studies have confirmed the validity and rationality of such self-reported health variables [8,13,14,31,32,33,34,35,36,37]. Second, this study examined only the income and psychological effects, but neglected other potential explanations at the societal or community level. Indeed, no data are available to identify those mechanism in this survey, with the investigation of these issues left for future research. Finally, since the study was based on cross-sectional data, we are not able to examine the causal relationship between the land expropriation and health. These should all be addressed in further studies.

## Figures and Tables

**Table 1:** Variable descriptions and descriptive statistics.

Variable	Description	Mean	S.D.
Health	Self-reported health status of respondents (1 = very poor; 2 = poor; 3 = fair; 4 = good; 5 = very good).	3.823	1.066
Land-lost	Land lost status (1 = lost land; 0 = have not lost land)	0.304	0.460
Gender	Gender (1 = male; 0 = female)	0.505	0.500
Age	Age (years)	45.001	17.932
Marriage	Martial statues (1 = have a spouse; 0 = divorced, unmarried or widowed)	0.806	0.395
Hukou	Hukou status (1 = Agricultural registered permanent residence; 0 = others)	0.817	0.387
Education	Education level declared (in years of schooling)	7.927	5.528
Nrinsurance Whether to participate New Rural Cooperative	Medical Scheme (1 = yes, 0 = other)	0.767	0.423
Fscale	Family size and scale (number of people)	4.116	1.337
Fincome	Family income per capita per year (ten thousand yuan)	0.304	0.629
Fland	Family farmland per capita (mu)	0.638	0.601
Mexpenses	Medical expenses per capita last year (ten thousand yuan)	0.046	0.208
Houseprice	Family house marketing value (ten thousand yuan)	10.226	16.210

Notes: S.D. = standard deviation.

**Table 2:** Results: ordered probit model estimating health status.

Variable	Model 1	Model 2	Model 3	Ordered Logit
Landlost	−0.158 (−3.67) ***	−0.114 (−2.49) **	−0.109 (−2.02) **	−0.180 (−1.96) **
Gender	0.128 (3.67) ***	0.145 (4.09) ***	0.130 (3.46) ***	0.225 (3.53) ***
Age	−0.0214 (−19.28) ***	−0.0209 (−18.24) ***	−0.0198 (−15.99) ***	−0.0338 (−15.65) ***
Marriage	0.0693 (1.51)	0.0758 (1.61)	0.0619 (1.24)	0.126 (1.48)
Hukou	−0.310 (−5.93) ***	−0.348 (−6.05) ***	−0.298 (−4.73) ***	−0.535 (−4.99) ***
Education	0.0277 (7.73) ***	0.0293 (7.92) ***	0.0313 (7.89) ***	0.0496 (7.07) ***
Nrinsurance		0.0678 (1.31)	0.0961 (1.73) *	0.153 (1.64)
Mexpenses		−0.392 (−10.79) ***	−0.455 (−11.04) ***	−0.852 (−10.00) ***
Fscale			0.0315 (2.13) **	0.0619 (2.45) **
Fincome			0.128 (4.26) ***	0.341 (4.17) ***
Fland			−0.00972 (−0.27)	−0.0504 (−0.83)
Houseprice			0.00323 (2.44) **	0.00431 (1.90) *
Log likelihood	−5098.4782	−4884.5761	−4374.0634	−4378.4682
Prob > *χ*^2^	0.0000	0.0000	0.0000	0.0000
Pseudo *R*^2^	0.0646	0.0767	0.0805	0.0795
Observations	3945	3820	3448	3448

Notes. (1) The cut points of the models are not reported in the table; (2) Figures in parentheses are *t*-statistics; (3) The symbols *, ** and *** indicate statistical significance at the 10%, 5% and 1% levels, respectively.

**Table 3:** Marginal effect of ordered probit estimations of health status.

Variables	Very Poor	Poor	Fair	Good	Very Good
Landlost	0.0023	0.0173	0.0212	−0.0022	−0.0385
Gender	−0.0025	−0.0200	−0.0254	0.0017	0.0461
Age	0.0004	0.0031	0.0039	−0.0003	−0.0071
Marriage	−0.0013	−0.0097	−0.0120	0.0012	0.0218
Hukou	0.0047	0.0412	0.0595	0.0045	−0.1100
Education	−0.0006	−0.0048	−0.0061	0.0004	0.0111
Nrinsurance	−0.0020	−0.0152	−0.0186	0.0020	0.0338
Mexpenses	0.0088	0.0699	0.0891	−0.0059	−0.1620
Fscale	−0.0006	−0.0048	−0.0062	0.0004	0.0112
Fincome	−0.0025	−0.0198	−0.0252	0.0017	0.0458
Fland	0.0002	0.0015	0.0019	−0.0001	−0.0035
Houseprice	−0.0001	−0.0005	−0.0006	0.0000	0.0011

**Table 4:** Robustness of estimating health status: adding some other control variables.

Variables	Model 5	Model 6	Model 7
Landlost	−0.112 (−2.03) **	−0.113 (−2.04) **	−0.115 (−2.07) **
Businesse	0.122 (2.70) ***	0.122 (2.70) ***	0.126 (2.78) ***
Expendnutri		0.274 (1.91) *	0.269 (1.87) *
Communistpm			0.0969 (1.14)
Observations	3448	3448	3448

Notes. (1) Figures in parentheses are *t*-statistics; (2) The symbols *, ** and *** indicate statistical significance at the 10%, 5% and 1% levels, respectively; (3) Other control variables used in Model 3 such as gender, age and marriage, etc. were all controlled.

**Table 5:** Robustness of estimating health status: the alternative measures.

Variables	Model 8 (Probit)	Model 9 (Logit)
Landlost	−0.274 (−3.20) ***	−0.474 (−3.02) ***
Observations	3448	3448

Notes. (1) Figures in parentheses are *t*-statistics; (2) The symbols *, ** and *** indicate statistical significance at the 10%, 5% and 1% levels, respectively; (3) Other control variables used in Model 3 such as gender, age and marriage etc. were all controlled.

**Table 6:** Robustness of estimating health status: changes in the sample range.

Variables	Model 10	Model 11		Model 12	Model 13
	(Under 65)	(Rural hukou)	(Urban Hukou)	(Under the line)	(Above the Line)
Landlost	−0.133 (−2.26) **	−0.103 (−1.70) *	−0.097 (−0.79)	−0.123 (−1.74) *	−0.153 (−1.79) *
Observations	2927	2768	680	2001	1447

Notes. (1) Figures in parentheses are *t*-statistics; (2) The symbols *, ** and *** indicate statistical significance at the 10%, 5% and 1% levels, respectively; (3) Other control variables used in Model 3 such as gender, age and marriage etc. were all controlled.

**Table 7:** Mechanism affecting health status: the effect of the land expropriation on the different incomes.

Variables	Agriculture-Related	Non-Agriculture	Transfer	Total
Landlost	−0.354 (−5.87) ***	−0.037 (−0.62)	0.294 (3.71) ***	0.048 (0.76)
Observations	2525	1365	2743	3448

Notes. (1) Figures in parentheses are t-statistics; (2) The symbols *, ** and *** indicate statistical significance at the 10%, 5% and 1% levels, respectively; (3) Other control variables used in Model 3 such as gender, age and marriage etc. were all controlled.

**Table 8:** Mechanism affecting health status: gender differences.

Variables	Model 14 (Male)	Model 15 (Female)
Landlost	−0.0181 (−0.24)	−0.205 (−2.67) ***
Observations	1745	1703

Notes. (1) Figures in parentheses are t-statistics; (2) The symbols *, ** and *** indicate statistical significance at the 10%, 5% and 1% levels, respectively; (3) Other control variables used in Model 3 such as age and marriage etc. are all controlled.

## Supplementary Materials

The following are available online at https://www.mdpi.com/1660-4601/16/16/2934/s1, Table S1: Correlation matrix of all variables.
