# Supplementary material for: Effect of Land Expropriation on Land-Lost Farmers’ Health: Empirical Evidence from Rural China
Source: Int J Environ Res Public Health. 2019 Aug 15;16(16):2934. doi: 10.3390/ijerph16162934 (PMC6720733; doi:10.3390/ijerph16162934)
Supplement: Supplementary file 1 [file ijerph-16-02934-s001.pdf]

Supplementary material:

Table S1. Correlation matrix of all variables.

Variables	Health	Landlost	Gender	Age	Marriage	Hukou	Education
Health	1						
Landlost	−0.0079	1					
Gender	0.0512 *	−0.014	1				
Age	−0.3851 *	−0.0181	0.02	1			
Marriage	−0.0438 *	0.0350 *	−0.0311 *	0.2213 *	1		
Hukou	−0.0874 *	−0.4949 *	−0.0158	0.0008	−0.0213	1	
Education	0.2877 *	0.0391 *	0.0845 *	−0.4785 *	0.0719 *	−0.0688 *	1
Nrinsurance	−0.026	−0.4852 *	0.0127	0.018	0.0433 *	0.5285 *	0.0083
Mexpenses	−0.3263 *	0.0498 *	−0.0348 *	0.1835 *	0.0326 *	0.0282	−0.1246 *
Fscale	0.0837 *	0.0033	−0.0292	−0.1743 *	0.0487 *	0.0145	0.0304
Fincome	0.1688 *	−0.0172	0.1387 *	−0.2017 *	0.0476 *	0.0452 *	0.2431 *
Fland	−0.0475 *	−0.5675 *	0.0353 *	0.0717 *	−0.0422 *	0.4953 *	−0.0283
Houseprice	0.1105 *	0.4680 *	−0.0148	−0.0709 *	0.0407 *	−0.4134 *	0.0971 *
**Variables**	**Nrinsurance**	**Mexpenses**	**Fscale**	**Fincome**	**Fland**	**Houseprice**	
Nrinsurance	1						
Mexpenses	0.0142	1					
Fscale	0.0002	−0.0042	1				
Fincome	0.0710 *	−0.0587 *	−0.1452 *	1			
Fland	0.4396 *	−0.0727*	−0.1304 *	0.0869 *	1		
Houseprice	−0.3695 *	−0.0289	0.1128 *	0.0386 *	−0.4559 *	1	

Notes. * indicates statistical significance at the 5% level.
